# Strategies to improve male involvement in PMTCT Option B+ in four African countries: a qualitative rapid appraisal

**DOI:** 10.3402/gha.v9.33507

**Published:** 2016-11-07

**Authors:** Donela Besada, Sarah Rohde, Ameena Goga, Nika Raphaely, Emmanuelle Daviaud, Vundli Ramokolo, Vuyolwethu Magasana, Nobuntu Noveve, Tanya Doherty

**Affiliations:** 1 Health Systems Research Unit, South African Medical Research Council, Cape Town, South Africa; 2 Health Systems Research Unit, South African Medical Research Council, Pretoria, South Africa; 3 Department of Paediatrics, University of Pretoria, Pretoria, South Africa; 4 Gender and Health Research Unit, South African Medical Research Council, Pretoria, South Africa; 5 School of Public Health, University of the Witwatersrand, Johannesburg, South Africa; 6 Health Systems Research Unit, South African Medical Research Council, Durban, South Africa; 7 School of Public Health, University of the Western Cape, Cape Town, South Africa

**Keywords:** PMTCT, male partner involvement, Option B+, community health cadres, Qualitative rapid appraisal

## Abstract

**Background:**

The World Health Organization recommends that antiretroviral therapy be started as soon as possible, irrespective of stage of HIV infection. This ‘test and treat’ approach highlights the need to ensure that men are involved in prevention of mother-to-child HIV transmission (PMTCT). This article presents findings from a rapid appraisal of strategies to increase male partner involvement in PMTCT services in Uganda, Democratic Republic of Congo, Malawi, and Côte d'Ivoire in the context of scale-up of Option B+ protocol.

**Design:**

Data were collected through qualitative rapid appraisal using focus groups and individual interviews during field visits to the four countries. Interviews were conducted in the capital city with Ministry of Health staff and implementing partners (IPs) and at district level with district management teams, facility-based health workers and community health cadres in each country.

**Results:**

Common strategies were adopted across the countries to effect social change and engender greater participation of men in maternal, child and women's health, and PMTCT services. Community-based strategies included engagement of community leaders through dialogue and social mobilization, involving community health workers and the creation and strengthening of male peer cadres. Facility-based strategies included provision of incentives such as shorter waiting time, facilitating access for men by altering clinic hours, and creation of family support groups.

**Conclusions:**

The approaches implemented at both community and facility levels were tailored to the local context, taking into account cultural norms and geographic regional variations. Although intentions behind such strategies aim to have positive impacts on families, unintended negative consequences do occur, and these need to be addressed and strategies adapted.

A consistent definition of ‘male involvement’ in PMTCT services and a framework of indicators would be helpful to capture the impact of strategies on cultural and behavioral shifts. National policies around male involvement would be beneficial to streamline approaches across IPs and ensure wide-scale implementation, to achieve significant improvements in family health outcomes.

## Introduction

There is increasing global recognition that involvement of men in reproductive health policy and service delivery is vital to ensure good health outcomes for men, women, and children. Traditionally, prevention of mother-to-child HIV transmission (PMTCT) programs has largely focused on women; however, there is strong evidence that the involvement of partners in women's reproductive health can have a significant impact on utilization of services such as antenatal care (ANC) and facility-based delivery, increasing uptake of HIV testing, enrollment into treatment, and retention of clients in PMTCT services (1, 2). The increased focus on couple counseling for HIV testing has improved communication and collective decision-making for treatment within households conferring benefits not only to the mother and her HIV-exposed children but also to her partner (3, 4).

Across many cultures, men are key decision-makers on issues affecting their wives and children particularly in sub-Saharan Africa where reproductive health decisions are greatly influenced by male partners. In this region, male partner involvement in PMTCT is generally low due to lower general utilization of health services among men compared with women, poor male knowledge of PMTCT, male reluctance to learn their HIV status, clashes between ANC/PMTCT timings and men's working hours, and women's hesitation to get tested alongside their partners due to fear of domestic violence if found to be infected (5).

With the shift to antiretroviral therapy (ART) for life regimens for pregnant and breastfeeding women regardless of CD4 count (referred to as World Health Organization [WHO] Option B+) (6) and the recent recommendation by WHO to ‘test and treat’(7), removing all limitations on eligibility for ART among people living with HIV, the imperative to involve men in PMTCT services becomes greater. As women are usually the first to be tested through ANC attendance, failure to use the opportunity of ANC as an entry point for testing men will severely limit the scale-up of ‘test and treat’ approaches and may hinder the long-term retention and adherence of families in care (7).

This article presents findings from a rapid appraisal of strategies to increase male partner involvement in PMTCT services in Uganda, Democratic Republic of Congo (DRC), Malawi, and Côte d'Ivoire in the context of scale-up of Option B+ protocol. It presents qualitative findings from individual interviews and focus group discussions (FGDs) with health managers, implementing partners (IPs), facility staff, and community health cadres in each country, accompanied by a desk review of relevant reports and policy documents. Identification of differences and commonalities in the approaches adopted across the four countries assists in the design of integrated and effective strategies to increase male partner involvement in PMTCT services.

## Methods

### Study design

This descriptive qualitative study (8) by rapid appraisal (9, 10) was part of a midterm evaluation of the UNICEF-supported Optimizing HIV Treatment Access (OHTA) for pregnant and breastfeeding women initiative. The initiative, funded by Sweden and Norad (The Norwegian Agency for Development Cooperation) through UNICEF, was undertaken in four countries (Malawi, Uganda, the DRC, and Côte d'Ivoire) between 2013 and 2015 in partnership with several IPs and their local agencies (11).

The OHTA initiative aimed to support the transition to antiretroviral treatment for life (Option B+) protocol for PMTCT, in the case of the DRC and Côte d'Ivoire, and to optimize delivery and increase demand in Uganda and Malawi, which were early adopters of the policy (12). OHTA focused on strengthening community–facility linkages through establishment or strengthening of community-based cadres.

The midterm evaluation aimed to assess progress with implementation of the Option B+ protocol for PMTCT in the four OHTA-supported countries including strategies to increase male partner involvement in the context of Option B+ scale-up.

### Data collection

Qualitative data were gathered through rapid appraisal (9, 10) during country field work and through a desk review of relevant documents (annual project reports from UNICEF and IPs, annual country reports to UNICEF, national PMTCT strategic plans, national male involvement in Reproductive, maternal, newborn, and child health (RMNCH) strategy documents, and academic published literature). Field visits, of approximately 12 days per country, by a mixed skill team of 3–4 researchers, took place during June and July 2015. Potential organizations and individuals for key informant interviews and FGDs were identified through a desk review process and were shared and amended in collaboration with UNICEF headquarters and country offices.

Although the compiled interview lists focused on gathering a wide range of opinions to ensure fair representation of how male partner involvement strategies were implemented, the terms of reference of the evaluation explicitly excluded interviews with clients (beneficiaries of services).

Semi-structured interview guides were developed for each category of respondents (Ministry of Health, IPs, district management team, facility-based health workers, and community cadres). These guides were reviewed by colleagues in UNICEF to ensure that they sufficiently captured the critical themes of the evaluation. Male involvement was explored through an opening question: ‘Is your facility/department/organization implementing any activities to encourage male involvement’. This was followed by probing questions based on findings from the desk review or prior interviews.

Each semi-structured interview was conducted by one or more researchers at the workplaces of the interviewees, and interviews lasted between 30 min and 1 h. Where necessary, especially with community cadres, a translator was used to explain the research aim and the consent process. Interviews were audio-recorded where permission was granted, and researchers took notes. Interviewees were given detailed explanation regarding the purpose of the interview and their rights, including the right not to participate. Signed informed consent from literate participants and recorded verbal consent from illiterate participants were obtained by the interviewer. None of the individuals approached refused to participate.

Each country visit included 3–4 days of meetings with UNICEF, Ministry of Health, IPs and other national-level stakeholders in the capital, followed by travel to outlying districts for visits to district management teams and health facilities. Table 1 shows the numbers of interviews undertaken in each country according to respondent type.

**Table 1:** Summary of participants

Type of interview	Participant category	Number of interviewees/focus group discussion participants
Malawi		
Data collection: 15–24 June 2015; districts visited: Lilongwe, Mzimba North, and Zomba		

Individual interviews	IP	2 female
	MoH	1 female, 2 male
	Multi-lateral agency	1 female, 1 male
	District management	5 male
	Facility-based health workers	2 female, 2 male
	Community-based health worker	1 female, 1 male
FGDs	IP	7 female, 4 male
	MoH	1 female, 2 males
	Multi-lateral agency	2 female; 3 male
	District management	1 female, 3 male
	Facility-based health workers	7 female, 5 male
	Community-based health workers (health surveillance assistants, Male Study Circles, M2M mentor mothers, Community advisory board)	10 groups (average size 9 individuals, mixed gender)

Côte d’ Ivoire		
Data collection: 19–31 July 2015; districts visited: Port-Bouet, Bouake Sud, and Daloa		

Individual interviews	IP	4 female, 6 male
	MoH	1 female, 3 male
	Multi-lateral agency	3 female, 3 male
	District management	1 female, 6 male
	Facility-based health workers	1 male
FGDs	IP	4 groups (average size 7, mixed gender)
	District management	1 group of 2 females and 4 males
	Facility-based health workers	1 group of 2 females and 1 male
	Community-based health workers (scouts, lay counselors, CHWs, traditional leaders)	3 groups (average size 8, mixed gender)

DRC		
Data collection: 8–19 June 2015; health zones visited in the Katanga Province: Kasenga, Kapemba, and Kisanga		

Individual interviews	IP	6 male
	MoH	2 female, 6 male
	Multi-lateral agency	4 female, 6 male
	District management	1 female, 3 male
	Facility-based health workers	2 female, 1 male
FGDs	IP	5 groups (average size 3, mixed gender)
	Facility-based health workers	1 group with 4 females
	Community-based health workers (relais communautaire, mentor mothers, peer educator)	4 groups (average size 4, mixed gender)

Uganda		
Data collection: 29 June to 19 July 2015; areas visited: Greater Kampala and nine districts across three regions (Bugiri, Kamuli, Kaliro, Isingiro, Bushenyi, Ibanda, Moroto, Kotido, Abim)		

Individual interviews	IP	6 female, 9 male
	MoH	2 female, 4 male
	Multi-lateral agency	2 male
	District management	30 female, 27 male
	Community-based health worker	2 female
FGDs	IP	1 group with 2 females and one male
	Facility-based health workers	2 groups (average size 4, mostly female)
	Community-based health workers (scouts, lay counselors, CHWs, traditional leaders)	13 groups (average size 5, mixed gender)

DRC, Democratic Republic of Congo; FGDs, focus group discussions; CHWs, Community health workers; IP, Implementing partner; M2M, Mothers 2 mothers; MoH, Ministry of Health.

### Data analysis

At the end of each visit, an initial overview of our insights was presented to UNICEF staff and Ministry of Health representatives. Later on, audio-recorded interviews were transcribed and/or translated and field notes summarized. We conducted a simple manifest analysis of the qualitative material (8, 13), as this was not an ethnographic study, and we were simply interested in documenting what was happening and participants’ experiences, rather than trying to understand the deeper meaning of the experience.

We analyzed the data both deductively and inductively (14). Deductively, we sought to find answers to predefined questions (e.g. how are men involved in PMTCT in this district?). Inductively, we tried to understand what new information and insights could be gleaned from the interviews and our desk review.

The analysis was based on the typed interview and focus group notes as well as reflections from the field. Country material was reviewed by each country team. We annotated our reflections while reading and came together to discuss, compare, and critique our insights. Data were then electronically (using a word processor) grouped into categories, the results of which are reported in narrative form in this article.

### Ethics approval

This study received ethical approval from the South African Medical Research Council (EC014-4/2015), and permission was received from each of the following authorities in the four countries: Malawi: Director of the HIV & AIDS Department in the National Ministry of Health; Uganda: Higher Degrees, Research and Ethics Committee, College of Health Sciences, School of Public Health, Makerere University; Côte d'Ivoire: The President, National Committee of Research and Ethics, Ministry of Health; DRC: The Clinical Director, National AIDS Control Programme, Ministry of Health.

## Results

### Strategies to increase male partner involvement in PMTCT Option B+

**Fig. 1:**
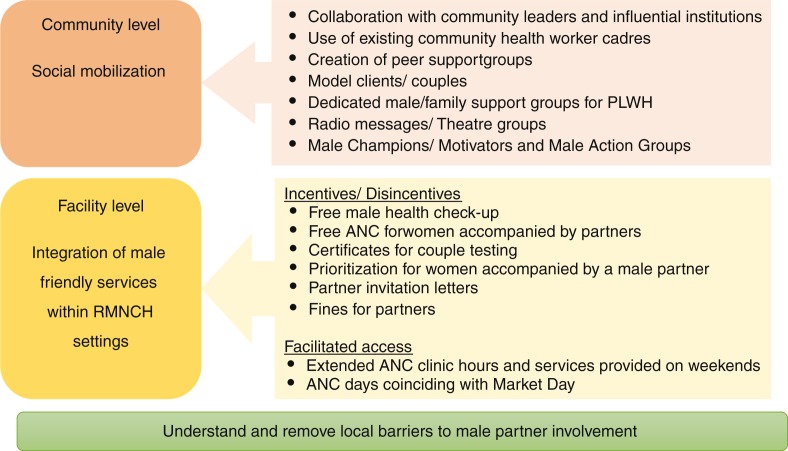
Strategies to increase male partner involvement in PMTCT. RMNCH, Reproductive, maternal, newborn, and child health; PLWH, people living with HIV/AIDS; ANC, antenatal care.

Interviews and FGDs identified common strategies adopted across the countries to effect social change and engender greater participation of men in RMNCH, and PMTCT services, which can be broadly grouped into two main categories: community-based strategies and facility-based strategies (Fig. 1).

### Community-based strategies: social mobilization through community dialogue and sensitization

#### Engagement of influential community leaders and community-based health cadres

Community dialogue and sensitization was one of the most commonly discussed male partner involvement strategies in key informant interviews. Community and religious leaders and influential figures were actively involved in the mobilization process as they command, respect, and influence, especially in rural communities.

In Uganda, the collaboration of village elders and community leaders in the elimination of mother-to-child transmission (eMTCT) of HIV was described as instrumental in initiating a shift in the population's attitude toward the role of men in ANC and eMTCT, and in gaining trusted access to communities. In nomadic communities in North-East Uganda, District Health Teams with support from IPs work to engage village leaders, as one IP staff member explained:

We have involved the elders, because we believe they are influential in the communities and they are the ones able to talk to these men and urge them to come for services. We keep talking to them. (IP, Uganda)

Similar approaches were described in the DRC and Côte d'Ivoire:

The first strategy is lobbying community leaders, including religious leaders. We strengthened the capacity of those giving messages in the community through HIV training. (Programme National pour la Lutte contre le SIDA (PNLS), DRC)

We try to involve the community leaders/chiefs to help them understand what it (PMTCT) is about and what is expected from them. They take part in the activities, they participate in meetings and convey information to the community. We have a committee for elimination of mother to child transmission. We put this in place in all health areas linked to the project (OHTA) – the ASCs (community health workers), lay counsellors, head of youth, women's associations, and village chiefs meet each month and plan mass sensitization activities on the importance of ANC, family practices and male involvement. (Nurse, Côte d'Ivoire)

Additionally, efforts have been directed toward fostering partnerships between community leaders and youth groups. In Côte d'Ivoire, local scout and youth groups work alongside elder community leaders to change attitudes in urban communities, educating men and youth about HIV prevention, the importance of getting tested and supporting their partners during ANC/PMTCT. Furthermore in Côte d'Ivoire, informants described the use of radio and local theatre to model scenarios of locally relevant cultural dynamics that influence attitudes to male partner involvement as one district manager described:

We have used local radio sessions to encourage men to come get tested with their wives. For example, there was one program (skit) where a woman tells her husband to get tested and he gets angry in beginning, but then she talks to him and explains the benefits and he changes his attitude in the end. (District Manager, Côte d'Ivoire)

Alongside collaboration with influential leaders, community health workers (CHWs), peer supporters, and other community cadres were trained to sensitize communities, families, and individuals on the importance of male partner involvement as one PMTCT coordinator from Malawi described:

Culturally male is the head, so if we involve them success would come. Also local leaders – they talk about these issues during their meetings, even for stigma – women would be able to take their treatment better. (PMTCT/ART Coordinator, Malawi)

In Côte d'Ivoire, CHWs were trained to sensitize communities on the importance of male partner involvement, as one district manager described:

Once they (CHWs) are trained they go into the field and sensitize women and partners to encourage them to visit health facilities. In terms of sensitization, many topics are covered including ANC, need for counseling and testing, need for population to accept their result and to go onto treatment, and skilled birth attendance and for staying into care until child is tested. (District manager, Côte d'Ivoire)

#### Creation and strengthening of male peer cadres

In Malawi and Uganda, dedicated male peer cadres have been established. In Malawi, an IP created Male Study Circles (MSCs), with volunteers called *male motivators*, who meet to discuss and learn about sexual and reproductive health, the importance of PMTCT, HIV prevention and family planning. Members are instrumental in educating other men in their communities on the importance of male partner involvement.

We move from village to village telling the husbands that their wives should be getting help from health centres. We also escort them to the health centres so that they can get help [and] emphasize that husbands should be taking part. (MSC member, Malawi)

One MSC member in Malawi described the benefits he experienced from being a male motivator:

In the beginning we [men] were behind but now we can participate so that we take pregnant women to the antenatal clinic. Sometimes we even carry them on bicycles. We start the antenatal visits together. So since [IP name] came, it has helped us to be men who are aware of what we are doing. (MSC member, Malawi)

Uganda has a similar program of peer educators known as male champions who receive 4 days of training on community sensitization including how to conduct large community dialogue meetings as one international IP respondent explained:

We ask committees to identify male champions. So we've tried that out where you know the committees come and say okay we think this is a model man, he looks after his family well, seems to support his wife when she is pregnant and all this kind of things, and we have trained them, taught them how to provide health promotion, how that specific to encouraging fellow men, you know, to be more engaged in sexual reproductive health services. (IP, Uganda)

Male champions are supported through IPs who provide them with bicycles and a small stipend to undertake their work. Male champions are expected to conduct quarterly community dialogue meetings and health education talks in the facilities twice a week as one district health officer explained:

Males who were identified and trained were given bicycles to ease their work so that they could move; interact with males in the public places like markets, drinking places and so on and so forth. (District Health Officer, Uganda)

In addition to male champions, some men living with HIV serve as expert clients to provide peer support and lead by example in their communities. As one IP explained:

These men are chosen in a way that they have been able to support their wives right from the pregnancy to the HIV-free baby so we can trust them for follow up and education. (IP, Uganda)

In Côte d'Ivoire, HIV-positive ‘model couples’ lead by example and work in communities to show what could be gained by using the PMTCT services and being on antiretroviral treatment.

They (implementing partner) worked with community leaders and mentors and they had model men and women to show what could be gained by using the services and being on treatment. (Ministry of Health, Côte d'Ivoire)

### Facility-based strategies: integration of male friendly services within the maternal and child health setting

Across the four countries, IPs have worked with local health structures and communities to introduce changes at the facility level to encourage men to accompany and support their partners for ANC/PMTCT services. Approaches have included strategies to integrate male friendly sexual and reproductive health services into PMTCT, initiating family support groups, incentivizing participation, and facilitating access for couples at ANC services.

#### Incentives

Incentives include offering free health screening services for men, giving couples priority in the waiting queue, giving certificates to couples testing together, and removing financial barriers.

In Uganda, men were given free health screening services and deworming medication when they came for ANC with their partners. As one respondent described:

To make sure that this is attractive to men what they do is make sure they have … they screen the men for and their women of course for syphilis, they screen them for blood pressure, hypertension, diabetes and then where there are circumcision services they link them to circumcision services. (Multi-lateral agency, Uganda)

Across all four countries, facilities prioritize women accompanied by their partner for maternal health visits. In Côte d'Ivoire, the *my partner, my visa* initiative gives priority in the waiting queue to women who present at ANC with their partners. In Malawi, ‘the referral system by Male Study Circles assist the couples to get preferential treatment at the facility, thereby encouraging men to accompany their wives’ (MSC member, Malawi).

A similar approach has been taken in Uganda:

if a women comes with her husband, you know, the husbands, the men are usually impatient you know so those who come as couples are served first, they take priority before the men run away anyway because the man says oh I have to go and look for money, you know, those kind of things. So those who come first, who come as couples, are served first. (Multi-lateral agency in Uganda referring to activities in the South-West region).

In Uganda's East-Central and South-West regions, however, respondents reported that while this strategy improved couple testing rates, some women would opt to bring any male with them in order to fulfill the requirement for them to reduce their wait time. Examples of this include paying *boda boda* (local motorbike taxi) drivers to act as their partner, as one health worker described:

We have a days for couple testing, HIV testing and counselling whereby they expect two people to come, a couple to come, but though it has had a lot of resistance, men are difficult to come, so what these ladies always do, they take bicycle riders to come and claim it's their partner. Yes, so they come and they, somebody who brought them on the cycle, say this is my partner, but as we move along, we notice this is not the partner now. (Hospital in-charge, Uganda)

In some facilities in Uganda and Côte d'Ivoire, formal certificates are presented to couples to engender a sense of accomplishment in having tested together. In the DRC, some facilities give mothers’ invitation cards addressed to their partners to encourage partner testing:

Invitation cards, they are helping to make the man feel recognized as he receives a formal request for attendance from us rather than be only asked by his partners [to come]. (DRC, Health Worker)

RMNCH services remain fee-based in the DRC, and one PMTCT IP sought to encourage male partner involvement by reducing financial barriers, ‘paying for deliveries if women come with their partners and attend all four ANC visits […] in sites that have major problems’ (IP, DRC). In another district, an IP refunded transport expenses when men accompanied their partner for ANC visits or delivery. This incentive, while creating a significant increase in the male attendances, only lasted for a short period due to cost implications, and male attendances decreased back to previous levels.

Although measures to encourage male partner involvement are generally positive in their approach, in Malawi, punitive measures were introduced in some communities with local leadership establishing bylaws whereby women were sent home during ANC if not accompanied by a male partner or men were fined (a goat) if their wives did not deliver in a health facility, as one IP respondent described:

So this is now a fine that is put in place by the traditional leaders …. this guy had a wife for five months without taking her to ANC, please have a goat from him … so that we felt like that if we had that across the district possibly we would have seen a lot of change. (IP, Malawi)

#### Facilitating men's access to ANC/PMTCT services

Traditional clinic hours present a major barrier to attendance of working men at ANC. In response, some facilities have shifted or extended ANC clinic hours.

In the DRC, an increasing number of facilities in the Lubumbashi area offer early morning or evening ANC appointments as one CHW described: ‘men work and they can't go for testing because it is during working hours’ (Relais Communautaire, DRC).

In North-East Uganda, where the majority of the population live far from health centers providing PMTCT, facilities have synchronized ANC hours with trading times in nearby livestock markets to rationalize household transport expenditure, as one respondent from an IP explained:

to catch the men that come to buy and sell their items. So the first thing they do is move with their women to the health unit, have their antenatal attendance together and then even if they go now doing their own things they have been involved they have been tested they have been talked to and then also there are some male champions in some of those facilities that really encourage them (to remain involved). (IP, Uganda)

#### Facility-based family support groups

An important strategy for retaining HIV-positive pregnant and breastfeeding women in care is family support groups, which serve to engage men in Malawi and Uganda. Groups meet monthly at health facilities where women pick up their medications and receive treatment. Women are encouraged to bring their partners where both receive holistic medical and psychosocial support. Crucially, these groups, which include male champions or male expert clients, recognize the family as a unit collectively responsible for its health and provide a peer support network (described above) that extends out of the facility context.

One health worker described what the family support group is and how it functions:

The family support group model is, it's a model of care, its psychosocial based, we meet mothers who are HIV positive and their partners and their children. We meet here every month, once a month. We share, the mothers share testimonies, success stories, what has helped me to disclose to my partner, how to take medicines, they reassure each other internally we feel the feedback they are sharing and so we know they are appreciating the service they come together and it has bonded them together. The bonding has left us to fully form them easily and it has taught us to facilitate transitioning in the ART clinic, the general ART clinic for lifelong ART. (Health worker, Uganda)

## Discussion

This qualitative study describes various strategies to increase male partner involvement in PMTCT across four countries implementing ART for life protocols. These strategies fall into two broad areas: community-based strategies utilizing community mobilization and sensitization, and facility-based strategies aimed at incentivizing male participation, facilitating access for men and provision of services targeted toward men individually or as family-centered services.

Common barriers to male involvement in RMNCH in sub-Saharan Africa have been well documented (5, 15, 16); however, variation in local contextual and cultural settings requires a deeper analysis of causative factors for lack of men's involvement in PMTCT and ANC activities. The strategies described by respondents were often shaped by the local context. Extending ANC hours was used to increase involvement of working men in urban settings, whereas in sparsely inhabited rural districts, strategies that rationalize households’ transport expenditures through synchronizing antenatal appointments with events of interest to men were commoner approaches.

Shifting cultural attitudes and beliefs takes time and requires the development of culturally sensitive and informed strategies. While challenges around male involvement remain, there is increased awareness of the importance of male partner involvement through community sensitization and mobilization across countries. Increased male participation has been facilitated through the use of male champions, to deliver messages around benefits of male involvement, dispel myths and reconstruct cultural notions around the strength of men who participate in their partners’ health and lead by example.

Health services’ recognition of men independent of their partners, both through provision of particular health services and acknowledgement by facilities when they do participate, could be instrumental in increasing male involvement. However, sensitizing men to the importance of participating as a unit in the health of their partners and children is equally vital.

Formal adoption of a male involvement policy lends credibility to the importance of the approach in improving patient health outcomes and ensuring systematic implementation of strategies. In November 2014, the Ugandan Ministry of Health launched a national male involvement strategy to expedite eMTCT. The new directives were, in effect, a formalization of the efforts that donor agencies and their IPs had been making for years. Similar initiatives by other countries would be instrumental in global efforts to reduce pediatric transmission and impact of HIV on pregnant mothers and their partners.

Caution must, however, be exercised in implementing strategies that could lead to unintended consequences. Qualitative research in Malawi (17) has described how health workers may refuse service provision to women accessing ANC without their partners, and our interviews in Uganda describe women taking other men to the clinic such as taxi drivers to jump queues or avoid being scolded for attending alone.

Shifts in attitudes and behaviors are complex, and assessing progress in male partner involvement has proved hard to measure. Consequently, evidence for the strategies’ effectiveness is sparse. A Cochrane systematic review (18) found only one study from Tanzania, an invitation card intervention, for inclusion in the review and concluded that further rigorous research is needed to inform evidence-based recommendations.

Regarding routine national monitoring, couple testing during ANC is commonly used as a proxy indicator for determining shifts in levels of male partner involvement at country level (19). Using this single proxy overlooks the broader impact male participation has on reproductive health services, and additional indicators should be considered.

## Strengths and limitations

A key strength of this rapid appraisal study was the inclusion of four diverse countries in Southern, Central and West Africa, at different stages with implementation of PMTCT Option B+. The extent of male partner involvement activities in PMTCT and RMNCH in each country reflects this with Malawi and Uganda having more extensive approaches compared with the DRC and Côte d'Ivoire, which are at an earlier stage of implementation. Key informant interviews and FGDs were undertaken with a wide range of stakeholders in the countries, from national to community level.

A limitation is the field research by rapid appraisal during short country visits. Thus, the impressions presented must be regarded as a snapshot, raising questions for further exploration, particularly regarding the impact of the identified strategies on increasing male partner involvement and their potential for scale-up. This study did not explore the perceptions of service users, both men and women, toward the male partner involvement strategies. These would be important to address in future research.

## Conclusions

This research has described a variety of approaches to increase male partner involvement in the context of PMTCT Option B+ in four African settings. The approaches implemented at both community and facility levels were tailored to the local context, taking into account cultural norms and geographic regional variations. While intentions behind such strategies aim to have positive impacts on families, unintended negative consequences do occur, and these need to be addressed and strategies subsequently adapted.

A consistent definition of ‘male involvement’ in PMTCT services and a framework of indicators would be helpful to capture the impact of strategies on cultural and behavioral shifts. National policies around male partner involvement would be beneficial to streamline approaches across IPs and ensure wide-scale implementation, to achieve significant improvements in family health outcomes. Continued sharing within and across countries will enhance the scale-up of such approaches.

## Paper context

The ‘test and treat’ approach highlights the importance of male involvement in prevention of mother-to-child HIV transmission. Previous studies have shown the impact of male involvement on mothers’ retention in treatment. This multi-country qualitative rapid appraisal of strategies to increase male partner involvement describes facility-based and community-based strategies implemented in four African countries, tailored to local context and cultural norms. National policies around male involvement would help streamline approaches across districts and implementing partners and facilitate scale-up.
